# FGFR3 signaling and function in triple negative breast cancer

**DOI:** 10.1186/s12964-019-0486-4

**Published:** 2020-01-27

**Authors:** Nicole J. Chew, Elizabeth V. Nguyen, Shih-Ping Su, Karel Novy, Howard C. Chan, Lan K. Nguyen, Jennii Luu, Kaylene J. Simpson, Rachel S. Lee, Roger J. Daly

**Affiliations:** 1 Cancer Program, Biomedicine Discovery Institute, Monash University, Melbourne, VIC 3800 Australia; 2 Department of Biochemistry and Molecular Biology, Monash University, Melbourne, VIC 3800 Australia; 3 Sir Peter MacCallum Department of Oncology, The University of Melbourne, Melbourne, VIC 3010 Australia; 4 Victorian Centre for Functional Genomics, Peter MacCallum Cancer Centre, Melbourne, VIC 3000 Australia

**Keywords:** Receptor tyrosine kinase, Fibroblast growth factor receptor, Oncogene, Targeted therapy, Signal transduction

## Abstract

**Background:**

Triple negative breast cancer (TNBC) accounts for 16% of breast cancers and represents an aggressive subtype that lacks targeted therapeutic options. In this study, mass spectrometry (MS)-based tyrosine phosphorylation profiling identified aberrant FGFR3 activation in a subset of TNBC cell lines. This kinase was therefore evaluated as a potential therapeutic target.

**Methods:**

MS-based tyrosine phosphorylation profiling was undertaken across a panel of 24 TNBC cell lines. Immunoprecipitation and Western blot were used to further characterize FGFR3 phosphorylation. Indirect immunofluorescence and confocal microscopy were used to determine FGFR3 localization. The selective FGFR1–3 inhibitor, PD173074 and siRNA knockdowns were used to characterize the functional role of FGFR3 in vitro. The TCGA and Metabric breast cancer datasets were interrogated to identify FGFR3 alterations and how they relate to breast cancer subtype and overall patient survival.

**Results:**

High FGFR3 expression and phosphorylation were detected in SUM185PE cells, which harbor a FGFR3-TACC3 gene fusion. Low FGFR3 phosphorylation was detected in CAL51, MFM-223 and MDA-MB-231 cells. In SUM185PE cells, the FGFR3-TACC3 fusion protein contributed the majority of phosphorylated FGFR3, and largely localized to the cytoplasm and plasma membrane, with staining at the mitotic spindle in a small subset of cells. Knockdown of the FGFR3-TACC3 fusion and wildtype FGFR3 in SUM185PE cells decreased FRS2, AKT and ERK phosphorylation, and induced cell death. Knockdown of wildtype FGFR3 resulted in only a trend for decreased proliferation. PD173074 significantly decreased FRS2, AKT and ERK activation, and reduced SUM185PE cell proliferation. Cyclin A and pRb were also decreased in the presence of PD173074, while cleaved PARP was increased, indicating cell cycle arrest in G1 phase and apoptosis. Knockdown of FGFR3 in CAL51, MFM-223 and MDA-MB-231 cells had no significant effect on cell proliferation. Interrogation of public datasets revealed that increased FGFR3 expression in breast cancer was significantly associated with reduced overall survival, and that potentially oncogenic FGFR3 alterations (eg mutation and amplification) occur in the TNBC/basal, luminal A and luminal B subtypes, but are rare.

**Conclusions:**

These results indicate that targeting FGFR3 may represent a therapeutic option for TNBC, but only for patients with oncogenic FGFR3 alterations, such as the FGFR3-TACC3 fusion.

Video abstract.

## Background

Breast cancer accounts for 25% of all cancer and ranks as the second most common cancer in the world [1]. Triple negative breast cancer (TNBC) is the most aggressive subtype that represents approximately 10–20% of breast cancers and its oncogenic drivers are poorly understood [2, 3]. TNBC lacks expression of estrogen receptor (ER), progesterone receptor (PR) and human epidermal growth factor receptor-2 (HER-2) resulting in clinical resistance to endocrine and trastuzumab therapy [4]. Chemotherapy remains the only treatment option since targeted treatment strategies are lacking [5]. TNBC is associated with higher tumor grade, larger tumor size, higher metastasis rate, lymph node involvement and a median survival of 13 months after relapse [6–8]. To improve patient outcomes, we need to identify new therapeutic targets to build a platform for personalized treatment strategies.

Fibroblast growth factor receptors (FGFRs) are a family of four highly conserved transmembrane receptor tyrosine kinases (RTKs), comprising of FGFR1, FGFR2, FGFR3 and FGFR4 [9]. Activated FGFRs initiate intracellular signaling cascades involved in regulating a wide range of physiological processes such as cellular differentiation, proliferation, survival and migration, embryonic development and angiogenesis [10]. Aberrant FGFR signaling has been reported in many human cancers including breast cancer, colorectal carcinoma and endometrial carcinoma, and contributes to oncogenesis, tumor progression and resistance to anticancer therapies [11–13]. FGFR alterations have been reported in approximately 7.1% of cancers (most commonly in urothelial and breast cancer), with gene amplification being the most frequent FGFR aberration (66%), followed by mutation (26%) and rearrangement (8%) [14]. Given the oncogenic potential of FGFRs and their ‘druggability’, there has been considerable interest in developing targeted cancer therapies directed towards these receptors. Dovitinib, a multi-tyrosine kinase inhibitor with FGFR-inhibiting activity, induced tumor regression in patient-derived xenograft models exhibiting gene sets related to the FGFR signaling pathway, highlighting the latter as potential predictors for Dovitinib sensitivity [15]. Dovitinib is currently in phase 2 clinical trials and has demonstrated modest efficacy against lung squamous cell carcinomas harboring FGFR1 amplification [16]. BGJ398, a highly potent and selective pan-FGFR kinase inhibitor in clinical trials, has demonstrated antitumor activity in advanced cholangiocarcinoma patients with FGFR2 alterations [17] and promoted tumor reductions in FGFR1-amplified breast cancer patients [18]. Erdafitinib, an inhibitor of FGFR1–4, resulted in tumor shrinkage in an adrenal carcinoma patient with the FGFR3-TACC3 fusion [19]. Pemigatinib is another selective FGFR inhibitor that is currently under evaluation for its efficacy and safety in patients with urothelial carcinoma (NCT03011372).

FGFRs represent potential therapeutic targets in many human malignancies including breast cancer [20]. FGFR1 amplification on chromosome 8p11–12 is the most common FGFR1 alteration [21, 22], occurring in 14% of breast cancers and 16–27% of luminal B breast cancer, where it is associated with poor prognosis, shorter overall survival and resistance to endocrine therapies [23–25]. FGFR1 amplification is also an independent negative prognostic factor in gastric cancer, lung squamous cell carcinoma and TNBC [26–28]. Knockdown of FGFR1 expression in a FGFR1-overexpressing TNBC cell line MDA-MB-231 significantly reduced cell migration [28] and knock-out of FGFR1 reduced primary tumor growth and metastasis in a mouse mammary tumor model [29]. FGFR2 amplification is also a common FGFR aberration, occurring in 5–10% of breast cancers and 4% of TNBCs, and FGFR2 signaling drives resistance to Tamoxifen in ER+ disease [30, 31]. Knockdown of FGFR2 significantly reduced cell survival in the TNBC cell line MFM223 and this cell line also showed substantial sensitivity to the FGFR inhibitor PD173074 [30]. In breast cancer, high FGFR2 expression is significantly associated with tumor size and metastasis, shorter overall survival and lower disease-free survival rates [32]. Expression of autocrine FGF2 is associated with the basal/TNBC subtype of breast cancer cell lines and primary breast cancers, and in the former, confers sensitivity to PD173074 [33].

While the roles of FGFR1 and FGFR2 in breast cancer have been studied in considerable detail, FGFR3 remains poorly characterized in this setting. Molecular screening via segmental transcript analysis identified a FGFR3-TACC3 fusion in a primary TNBC specimen and TNBC cell line, SUM185PE [34]. In this fusion, the FGFR3 kinase domain is fused to the upstream region of the coiled-coil domain of transforming acidic coiled-coil 3 (TACC3) protein [34, 35]. FGFR3-TACC3 fusions also occur in other cancers, such as glioblastoma (3 out of 97 tumors examined, 3.1%), bladder cancer (2 of 43 bladder cancer cell lines, 4.7%) and nasopharyngeal carcinoma (4 out of 159 patients, 2.5%) [35–37]. The presence of the coiled-coil domain of TACC3 enhances dimerization of the fusion protein, thus activating the FGFR3 tyrosine kinase [38]. The presence of the FGFR3-TACC3 fusion increases cell proliferation and tumor formation in vivo [35], but confers sensitivity to specific FGFR inhibitors, indicating an oncogenic addiction to the fusion [37, 39, 40].

Previously, we utilized MS to compare the tyrosine phosphorylation profiles of luminal breast cancer and TNBC cell lines. This identified a prominent Src family kinase signaling network in TNBC and highlighted multiple kinases for further evaluation as therapeutic targets and biomarkers [41]. In this study, we applied this approach to a large panel of TNBC cell lines to interrogate this disease subtype in more detail and identify targets for personalized treatment. One potential target that emerged was FGFR3, and this was characterized in detail in this study.

## Materials and Methods

### Cell lines, cell culture and reagents

The BT549, BT20, DU4475, HCC38, HCC70, HCC1500, HCC1569, HCC1954, HCC1806, HCC1143, HCC1937, HS578T, MDA-MB-157, MDA-MB-436, MDA-MB-453, MDA-MB-231 and MDA-MB-468 cell lines were purchased from the American Type Culture Collection (ATCC; Manassas, VA, USA). CAL51, CAL148 and CAL851 cells were obtained from Deutsche Sammlung von Mikroorganismen und Zellkulturen (DSMZ) and CAL120 cells were a gift from Professor Elgene Lim from the Garvan Institute of Medical Research, Darlinghurst, NSW 2010, Australia. MFM223 cells were purchased from Sigma Aldrich. SUM185PE and SUM149PT cells were purchased from Asterand Bioscience. Cells were cultured in RPMI 1640 (Gibco) supplemented with 10% (v/v) FBS, 10 μg/mL insulin and 20 mM HEPES.

### Tyrosine phosphorylation profiling by mass spectrometry

To harvest proteins for mass spectrometry (MS) analysis, TNBC cell lines were cultured until 80% confluent, washed twice with ice cold phosphate-buffered saline (PBS), and lysed directly in the dish with lysis buffer (6 M guanidine hydrochloride, 50 mM Tris-HCl, 1 mM sodium orthovanadate, 2.5 mM sodium pyrophosphate, 1 mM b-glycerophosphate). Approximately 20 mg of lysate protein was reduced with 5 mM TCEP at 37 °C for 1 h and alkylated with iodoacetamide in the dark for 1 h. The samples were then diluted 1:4 with ammonium bicarbonate (25 mM) before digestion with a 1:200 LysC (Worthington) at room temperature (RT) for 4 h. Samples were further diluted 10x from the original volume before digested with a 1:100 trypsin (Promega) at 37 °C for 18 h. Tryptic digests were acidified with 10%TFA to pH 3 before desalting on a C18 column (Thermo Fisher Scientific) and elution with 0.1% TFA/40% ACN. Peptides were dried in a SpeedVac and reconstituted in 1.8 ml of IAP wash buffer (1% n-octyl-b-D-glucopyranoside, 50 mM Tris-HCl, 150 mM NaCl, pH 7.4). 50 μg each of P-Tyr-1000 (Cell Signaling Technology, 8954), P-Tyr-100 (Cell Signaling Technology, 9411), and P-Tyr-20 (BD Biosciences, 610,000) antibodies were coupled to 60 μL of sepharose beads slurry (Rec-Protein G, Zymed) and incubated overnight with peptide samples at 4 °C with gentle shaking. Immobilized antibody beads were washed three times with IAP buffer and further washed three times with water before elution with 110 μL of 0.15% TFA. Samples were then desalted on a C18 column (as described above) and evaporated to dryness in a SpeedVac. The dried peptides were reconstituted in 2% ACN/0.5% FA.

### Mass spectrometry analysis

Samples were analyzed on an UltiMate 3000 RSLC nano LC system (Thermo Fisher Scientific) coupled to an LTQ-Orbitrap mass spectrometer (LTQ-Orbitrap, Thermo Fisher Scientific). Peptides were loaded via an Acclaim PepMap 100 trap column (100 μm × 2 cm, nanoViper, C18, 5 μm, 100 Å, Thermo Fisher Scientific) and subsequent peptide separation was on an Acclaim PepMap RSLC analytical column (75 μm × 50 cm, nanoViper, C18, 2 μm, 100 Å, Thermo Fisher Scientific). For each liquid chromatography-tandem mass spectrometry (LC-MS/MS) analysis, 1 μg of peptides as measured by a nanodrop 1000 spectrophotometer (Thermo Fisher Scientific) was loaded on the pre-column with microliter pickup. Peptides were eluted using a 2 h linear gradient of 80% ACN/0.1% FA at a flow rate of 250 nL/min using a mobile phase gradient of 2.5–42.5% ACN. The eluting peptides were interrogated with an Orbitrap mass spectrometer. The HRM DIA method consisted of a survey scan (MS1) at 35,000 resolution (automatic gain control target 5e6 and maximum injection time of 120 ms) from 400 to 1220 m/z followed by tandem MS/MS scans (MS2) through 19 overlapping DIA windows increasing from 30 to 222 Da. MS/MS scans were acquired at 35,000 resolution (automatic gain control target 3e6 and auto for injection time). Stepped collision energy was 22.5, 25, 27.5% and a 30 m/z isolation window. The spectra were recorded in profile type.

### HRM-DIA data analysis

The DIA data were analyzed with Spectronaut 8, a mass spectrometer vendor-independent software from Biognosys. The default settings were used for the Spectronaut search. Retention time prediction type was set to dynamic indexed Retention Time (iRT; correction factor for window 1). Decoy generation was set to scrambled (no decoy limit). Interference correction on MS2 level was enabled. The false discovery rate (FDR) was set to 1% at peptide level. A peptide identification required at least 3 transitions in quantification. Quantification was based on the top 3 proteotypic peptides for each protein, normalized with the default settings, and exported as an excel file with Spectronaut 8 software [42]. For generation of the spectral libraries, DDA measurements of each sample were performed. The DDA spectra were analyzed with the MaxQuant Version 1.5.2.8 analysis software using default settings. Enzyme specificity was set to Trypsin/P, minimal peptide length of 6, and up to 3 missed cleavages were allowed. Search criteria included carbamidomethylation of cysteine as a fixed modification; oxidation of methionine; acetyl (protein N terminus); and phosphorylation of serine, threonine, and tyrosine as variable modifications. The mass tolerance for the precursor was 4.5 ppm and for the fragment ions was 20 ppm. The DDA files were searched against the human UniProt fasta database (v2015–08, 20,210 entries) and the Biognosys HRM calibration peptides. The identifications were filtered to satisfy FDR of 1% on peptide and protein level. The spectral library was generated in Spectronaut and normalized to iRT peptides.

### Cell lysis

Cells at 80% confluency were washed twice with ice cold 1x PBS then lysed with RIPA buffer (0.5% (w/v) sodium deoxycholate, 150 mM NaCl, 1% (v/v) NP40, 50 mM Tris˗HCl pH 8.0, 0.1% (w/v) sodium dodecyl sulfate (SDS), 10% (v/v) glycerol, 5 mM EDTA and 20 mM NaF), supplemented with 10 μg/mL aprotinin, 1 mM phenylmethane sulfonyl fluoride (PMSF), 10 μg/mL leupeptin, 1 mM sodium orthovanadate, 2.5 mM sodium pyrophosphate and 2.5 mM β-glycerophosphate prior to use. Lysed cells were collected and clarified by centrifugation at 21130 x *g* at 4 °C for 10 min, then the protein concentration was determined using a Pierce BCA protein assay kit (Thermoscientific) according to the manufacturer’s protocol.

### Western blotting

Protein lysates were subjected to Western blot analysis with antibodies. The following antibodies were purchased from Cell Signaling Technology: FGFR1 (9740), wildtype FGFR3 (4574), pan-phosFGFR (Y653, Y654) (3471), TACC3 (8069), AKT (4685), ERK (4695), pAKT (S473) (4058), pERK (T202, Y204) (4370), pFRS2 (Y436) (3861), PARP (9546), Rb (9313) and pRb (S780) (3590). The following antibodies were purchased from Santa Cruz Biotechnology: FGFR2 (sc-6930), FW FGFR3 (sc-13,121), FGFR4 (sc-136,988), pFGFR3 (Y724) (sc-33,041), FRS2 (sc-17,841), cyclin A (sc-53,227) and β-actin (sc-69,879). Two α-tubulin antibodies were purchased from Sigma-Aldrich (T5168) and from Abcam (ab6046).

### Immunoprecipitation

Protein lysates (2.5 mg) were incubated with 10 μg of the indicated antibodies overnight at 4 °C with gentle rotation. 40 μL of recombinant protein G-Sepharose 4B conjugate beads (Life Technologies, 101,242) was equilibrated in RIPA buffer were added to samples and incubated for 3 h at 4 °C with gentle rotation. Samples were centrifuged at 500 x *g* for 1 min at 4 °C and the unbound fraction transferred to a fresh microfuge tube. Beads were the washed thrice with RIPA buffer and centrifuged for 1 min at 500 x *g* at 4 °C and the supernatant removed. Immunoprecipitated proteins were then eluted using 2x sample loading buffer.

### Immunofluorescence and cell synchronization

SUM185PE cells seeded onto coverslips were fixed and permeabilized with PTEMF buffer (20 mM PIPES pH 6.8, 0.2% (v/v) Triton X 100, 10 mM EGTA, 1 mM MgCl_2_, 4% (v/v) PFA) 24 h post seeding for 20 mins. The samples were then blocked with 1% (w/v) bovine serum albumin for 1 h then immunostained with the indicated primary antibodies for 2 h followed by either anti-mouse Alexa Fluor 488 (Life Technologies, A21202) or anti-rabbit Alexa Fluor 555 (Life Technologies, A21428) for 1 h. All antibody incubations were performed at RT. Coverslips were mounted onto microscope slides with ProLong Gold Antifade Mountant with DAPI (Invitrogen). Cells were imaged 48 h later by immunofluorescence using a Nikon inverted confocal microscope. For cell synchronization, SUM185PE cells were synchronized at G1/S phase by 3 mM thymidine block for 18 h then released into media for 9 h. Next, the cells were then subjected to 3 mM thymidine block for another 15 h, released into media for 45 h and imaging was undertaken as above. Mitotic spindles were visualized by staining with rabbit anti-α-tubulin (Abcam, 6046) or mouse anti-α-tubulin (Sigma-Aldrich, T5168).

### Cell viability assays

For assays with siRNAs knockdown, SUM185PE cells were seeded into 96 well plates and cultured for 6 days, while CAL51, MFM223 and MDA-MB-231 were cultured for 4 days, with an 80% end point confluence for all the cell lines. Cell viability was determined using CellTiter96 Aqueous One Solution Cell Proliferation Assay (Promega) according to the manufacturer’s protocol. Absorbance was determined using the PHERAstar microplate reader (BMG LABTECH).

For assays with PD173074 treatment, SUM185PE cells and MDA-MB-468 cells were seeded into 6 well plates and cultured for 7 days with an 80% end point confluence. Cell numbers were obtained via direct cell counting. Cells were washed with 1x PBS then trypsinized at 37 °C in a 5% CO2 atmosphere until detachment. Trypsinized cells were then resuspended thoroughly in complete media to inhibit trypsin. Cells were stained with Trypan blue (EVS-1000, NanoTek), then transferred to an EVE cell counting slide (EVS-1000, NanoTek) and counted with the EVE automatic cell counter (EVE-MC-DEMO, NanoTek) according to the manufacturer’s protocol.

### PD173074 treatment

The selective small molecule inhibitor of FGFR1–3, PD173074 (Apex Biotech), was reconstituted in DMSO. For Western blotting, cells were treated with 5–1000 nM PD173074 for the indicated time before lysing in RIPA buffer. For viability assays, cells were treated with PD173074 24 h post seeding and viability determined at the indicated days.

### siRNA knockdown

In 96 well plate format, 7000 SUM185PE cells were reverse transfected with 0.15 μL of DharmaFECT1 (Dharmacon RNAi Technologies, Horizon Discovery). Media were changed 24 h later, replaced again at 96 h and the experiment ended at 144 h post transfection. 8000 CAL51 cells were reverse transfected with 0.1 μL of lipofectamine 3000 (Thermofisher Scientific), 5000 MDA-MB-231 cells with 0.1 μL of DharmaFECT4 (Dharmacon RNAi Technologies, Horizon Discovery) and 10,000 MFM223 cells with 0.1 μL of DharmaFECT3 (Dharmacon RNAi Technologies, Horizon Discovery). Media were changed 24 h later and the experiment ended at 96 h post transfection. In 6 well plate format, 360,000 SUM185PE cells, 300,000 CAL51 cells, 300,000 MFM223 cells and 90,000 MDA-MB-231 cells were reverse transfected with 3 μL of the corresponding lipid as previously mentioned. Media were changed 24 h later and the experiment ended at 72 h post transfection.

The FGFR3-TACC3 fusion and wildtype FGFR3 were knocked down together using ON-TARGETplus human FGFR3 set of 4 individual siRNAs labelled as FW 1–4 (Dharmacon RNAi Technologies, Horizon Discovery, Q-003133-00). Wildtype FGFR3 expression was knocked down using 3 individual custom FGFR3 siRNAs from Bioneer with the following sequence: GAGGAAAAGGCUGGUACAA (W1), CACAUGUCCAGCACCUUGU (W2) and GAUGCUGUGUAUAUGGUAU (W3). The ON-TARGETplus non-targeting SMARTpool (siOTP-NT) was used as the control (Dharmacon RNAi Technologies, Horizon Discovery, D-001810-10). All siRNAs were used at a final concentration of 20 nM.

### Quantification and statistical analysis

Quantification by densitometry was performed using ImageLab version 5.2.1 (Bio-Rad) and statistical *t*-tests were performed using GraphPad Prism 8 and Microsoft-Excel.

## Results

### Expression and phosphorylation of FGFRs and FGFR3-TACC3 fusion protein in TNBC cell lines

**Fig. 1:**
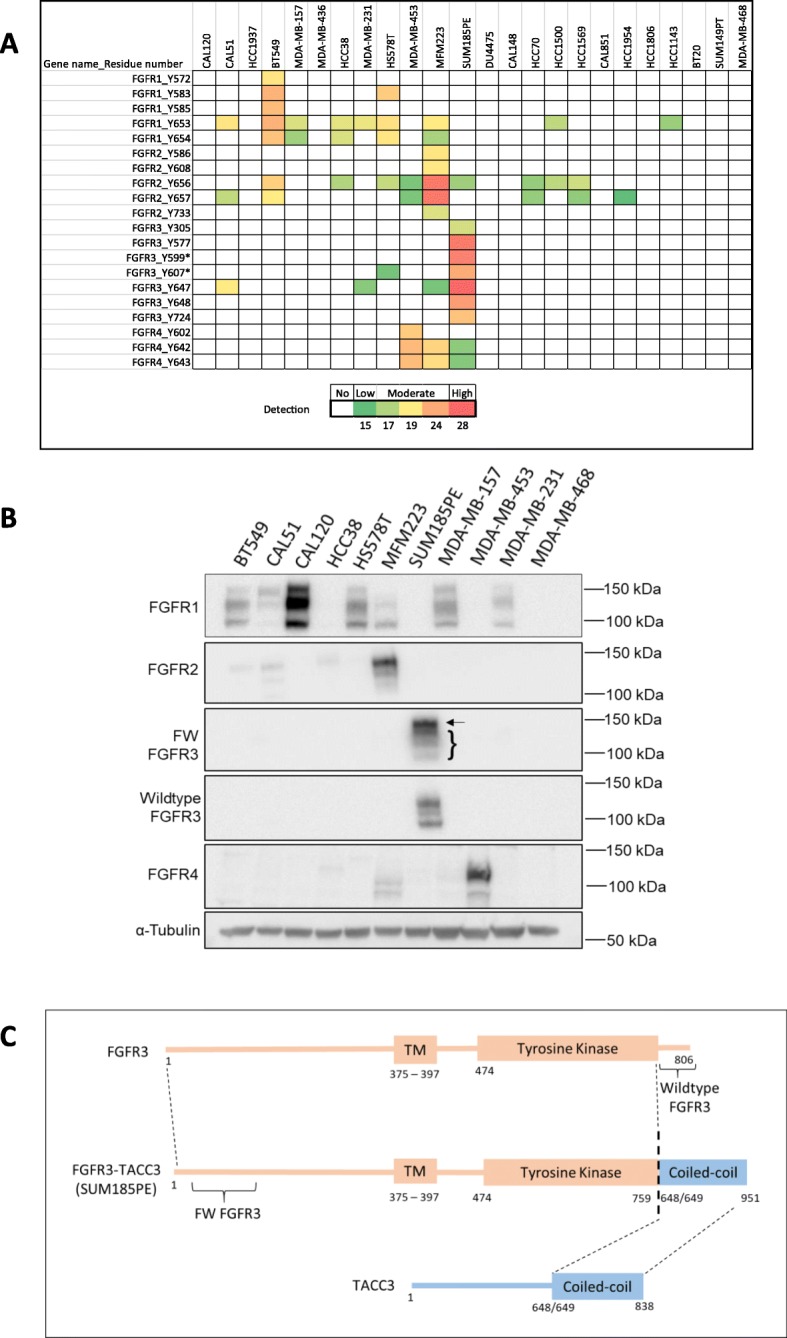
FGFR expression and phosphorylation signature in TNBC cell lines as determined by MS-based tyrosine phosphorylation profiling. **a**, Relative normalized abundance of FGFR1–4 phosphorylated tyrosine (pY) residues based on z-score across a panel of 24 TNBC cell lines. The z-scores of detectable tyrosine-phosphosites were obtained by subtracting the mean of all pY sites across the 24 TNBC cell line panel from the value for the pY site, and then dividing by the standard deviation of all 24 TNBC cell lines. The white box represents a non-detectable pY site. The asterisks indicate that FGFR3_Y599 is identical to FGFR1 (Y605) while FGFR3_Y607 is identical to FGFR2 (Y616), but the FGFR3 assignment is more likely given relative receptor expression levels. **b**, Characterization of FGFR1–4 expression in a panel of 11 TNBC cell lines. Cell lysates were Western blotted as indicated. Arrow indicates FGFR3-TACC3 fusion protein, bracket indicates wildtype FGFR3. **c,** Schematic of FGFR3-TACC3 fusion protein adapted from Shaver et al. (2016). The protein structure of wildtype FGFR3 is shown in pink and wildtype TACC3 is shown in blue. The grey dotted lines highlight the junction between FGFR3 and TACC3, which forms the FGFR3-TACC3 fusion protein in the SUM185PE cell line. FW FGFR3 antibody detects the region of FGFR3 between amino acids 25–124, recognising both wildtype FGFR3 and the FGFR3-TACC3 fusion protein. Wildtype FGFR3 antibody detects FGFR3 at the C-terminal region, only recognising wildtype FGFR3. TM = transmembrane

To identify potential therapeutic targets in TNBC, global MS-based phosphotyrosine profiling was undertaken. First, a data-dependent acquisition (DDA) workflow was used to generate a spectral library, with 2287 phosphotyrosine sites identified across the 24 TNBC cell lines. Then a hyper-reaction monitoring data-independent acquisition (HRM-DIA) workflow was utilized to quantitatively profile tyrosine phosphorylation patterns across this panel. Since FGFRs are implicated in cancer, including breast cancer, and represent candidate therapeutic targets, we extracted data for specific FGFR phosphorylation sites from this dataset (Fig. 1a, Additional file 2: Table S1). In addition, a panel of 11 TNBC cell lines was selected and subjected to Western blot analysis using selective FGFR antibodies (Fig. 1b). FGFRs resolve as a doublet (FGFR2 and FGFR4) or a triplet (FGFR1 and FGFR3) upon SDS-PAGE due to post-translational modifications (Fig. 1b). Overall, the results revealed high activation and expression of specific FGFRs, highlighting them as potential oncogenic drivers and therapeutic targets in TNBC.

Moderate FGFR1 phosphorylation was observed in BT549, CAL51, HS578T and MFM223 cells, and low phosphorylation in an additional 5 cell lines (Fig. 1a). High FGFR1 expression was detected by Western blotting in CAL120 cells and low to moderate levels in a further 6 cell lines (Fig. 1b). The results for the CAL120 cell line indicate that high FGFR1 expression may not be accompanied by detectable tyrosine phosphorylation (Fig. 1-1a-b).

High FGFR2 phosphorylation was detected in MFM223 cells, moderate phosphorylation in BT549 and low phosphorylation in an additional 9 cell lines (Fig. 1a). High FGFR2 expression was detected in MFM223 cells, and low expression detected in 3 cell lines (Fig. 1b). The results indicate that high FGFR2 phosphorylation correlates with high FGFR2 expression in MFM223 cells (Fig. 1-1a-b).

Moderate FGFR4 phosphorylation was detected in MDA-MB-453 and MFM223 cells (Fig. 1a), and low phosphorylation in SUM185PE cells (Fig. 1a). High and moderate FGFR4 expression was detected in the first two cell lines, respectively (Fig. 1b).

High FGFR3 expression and phosphorylation was detected in SUM185PE cells. In addition, moderate phosphorylation was detected in CAL51 cells and low phosphorylation in an additional 3 cell lines (Fig. 1-1a-b). The SUM185PE cell line harbors a FGFR3-TACC3 fusion [34], and interrogation of our phosphoproteomic dataset revealed that SUM185PE cells were the only TNBC cell line to exhibit tyrosine phosphorylation of TACC3, likely reflecting autophosphorylation of the fusion protein, and the TACC3 interactor CKAP5 (Additional file 2: Table S2 and Figure 1). To distinguish between the FGFR3-TACC3 fusion and the wildtype FGFR3, two antibodies were used (Fig. 1c). FW FGFR3 detects the region of FGFR3 between amino acid 25–124, thereby recognising both wildtype FGFR3 and the FGFR3-TACC3 fusion protein (detected as a slower migrating band above the wildtype FGFR3) (Fig. 1-1b-c). The wildtype-FGFR3 antibody is selective for this form of the receptor as the epitope localizes at the C-terminal region (Fig. 1-1b-c). The results indicate that SUM185PE cells express high levels of wildtype FGFR3 as well as the FGFR3-TACC3 fusion (Fig. 1b). The presence of both wildtype FGFR3 and an oncogenic form, FGFR3-TACC3 fusion in SUM185PE cells, apparent FGFR3 activation in other TNBC cell lines, and the lack of information regarding FGFR3 signaling and function in TNBC, led us to focus on this receptor.

### Tyrosine phosphorylation of wildtype FGFR3 and the FGFR3-TACC3 fusion in SUM185PE cells

**Fig. 2:**
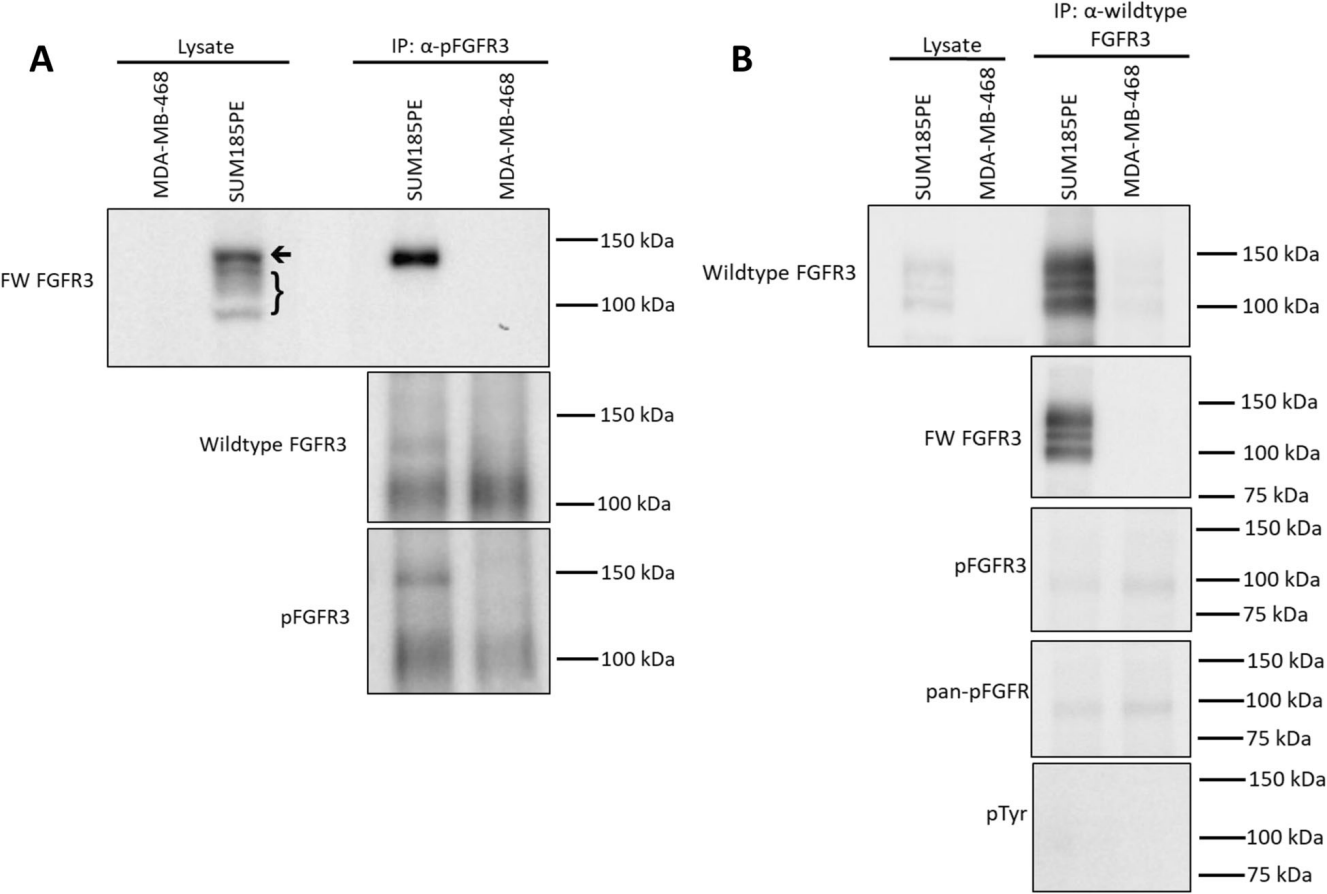
Characterization of FGFR3 phosphorylation in the SUM185PE cell line. **a**, Immunoprecipitation using a pFGFR3 antibody. SUM185PE and MDA-MB-468 (negative control) cell lysates were subjected to immunoprecipitation with the pFGFR3 antibody and then Western blotted with the indicated antibodies. The arrow indicates the FGFR3-TACC3 fusion protein and the bracket indicates wildtype FGFR3. **b**, Immunoprecipitation using the wildtype FGFR3 antibody. Cell lysates were subjected to immunoprecipitation with the wildtype FGFR3 antibody and blotted with the indicated antibodies

Since the SUM185PE cell line demonstrated high expression of both wildtype FGFR3 and the FGFR3-TACC3 fusion (Fig. 1b), accompanied by high FGFR3 phosphorylation (Fig. 1a), it was necessary to determine the contribution of the two receptor forms to this phosphorylation pattern. Tyrosine phosphorylated FGFR3 was enriched by immunoprecipitation using a selective antibody then blotted for FGFR3 using the two discriminatory antibodies (Fig. 2a). In this study, the MDA-MB-468 cell line with undetectable FGFR expression and phosphorylation (Fig. 1-1a-b) was used as a negative control. In the SUM185PE lysate enriched for tyrosine phosphorylated FGFR3, a band of the same mobility as the FGFR3-TACC3 fusion was readily detected when immunoblotted with the FW-FGFR3 antibody (Fig. 2a). A faint band was detected with the wildtype FGFR3 antibody (Fig. 2a). However, using this approach, wildtype FGFR3 may be co-purified in the pFGFR3 fraction, but not be directly tyrosine phosphorylated. To confirm the faint band detected in the wildtype FGFR3 blot in Fig. 2a, wildtype FGFR3 was enriched and blotted for phosphorylation using pFGFR3, pan-pFGFR and pTyr antibodies (Fig. 2b). No additional bands were observed in these blots compared to the negative control, indicating phosphorylation of wildtype FGFR3 was undetectable by this approach (Fig. 2b). These results indicate that the FGFR3-TACC3 fusion must contribute to the majority of phosphorylated FGFR3 in SUM185PE cells.

### The FGFR3-TACC3 fusion predominantly localizes to the cytoplasm and plasma membrane

TACC3 is a microtubule-associated protein that regulates mitotic spindle organization and stabilization, with the C-terminal coiled-coil domain of TACC3 mediating localization to the mitotic spindle [38, 43]. In glioblastoma, the FGFR3-TACC3 fusion was demonstrated to localize at the mitotic spindle poles in dividing cells, causing chromosomal segregation defects and triggering aneuploidy [35]. Furthermore, fractionation studies in MCF7 cells showed strong FGFR3-TACC3 fusion localisation to the nucleus [44]. However, a later study demonstrated that entry into the secretory pathway or plasma membrane localization was essential for cell transformation by the FGFR3-TACC3 fusion [45]. Furthermore, in HeLa cells, the FGFR3-TACC3 fusion was found to localize outside the spindle region in membrane vesicles, causing mitotic defects by removing wildtype TACC3 from the mitotic spindle [38]. These findings indicate that the localization and mechanism of FGFR3-TACC3 fusion may vary according to cancer type and cellular context. Consequently, it was important to address the subcellular localization of the FGFR3-TACC3 fusion in SUM185PE TNBC cells.

**Fig. 3:**
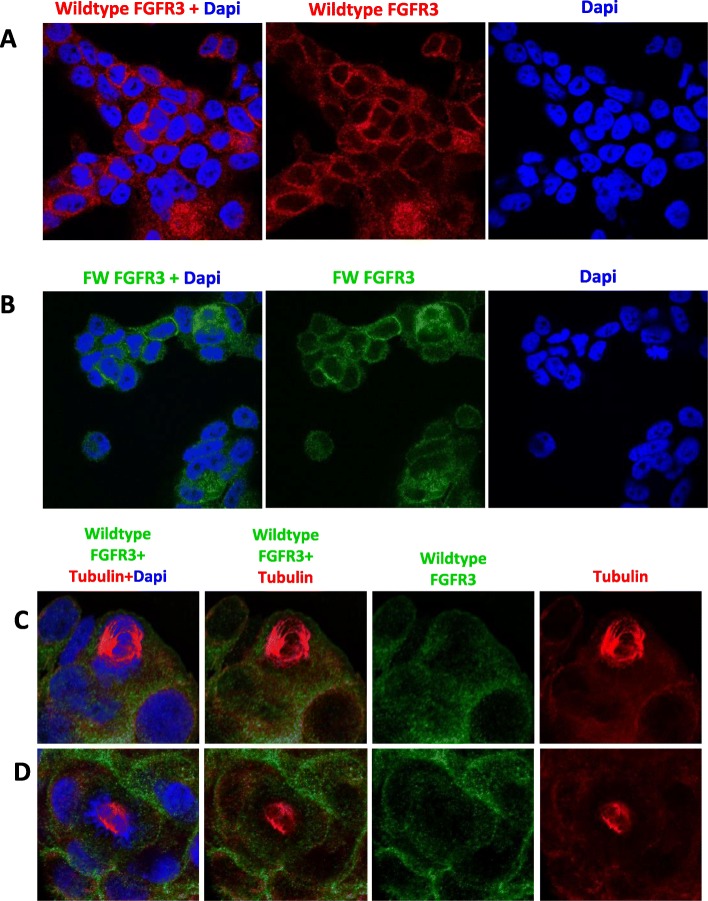
Localization of FGFR3-TACC3 fusion and wildtype FGFR3 by immunofluorescent staining in SUM185PE cells. SUM185PE cells were fixed and permeabilised then immunostained with **a**, wildtype FGFR3 antibody or **b**, FW FGFR3 antibody detecting both FGFR3-TACC3 fusion and wildtype FGFR3. Dapi was used to stain DNA of the cells. Images were obtained by confocal microscopy and are representative of 3 biological replicates, each involving analysis of at least 10 cells. **c** and **d**, representative images for immunostaining with the wildtype FGFR3 antibody in mitotic SUM185PE cells. For spindle visualisation, SUM185PE cells were treated with 3 mM of thymidine to halt cell cycle progression at the G1/S phase, and then released into complete media to allow cells to undergo mitosis. Tubulin immunostaining was used to visualize the mitotic spindle

**Fig. 4:**
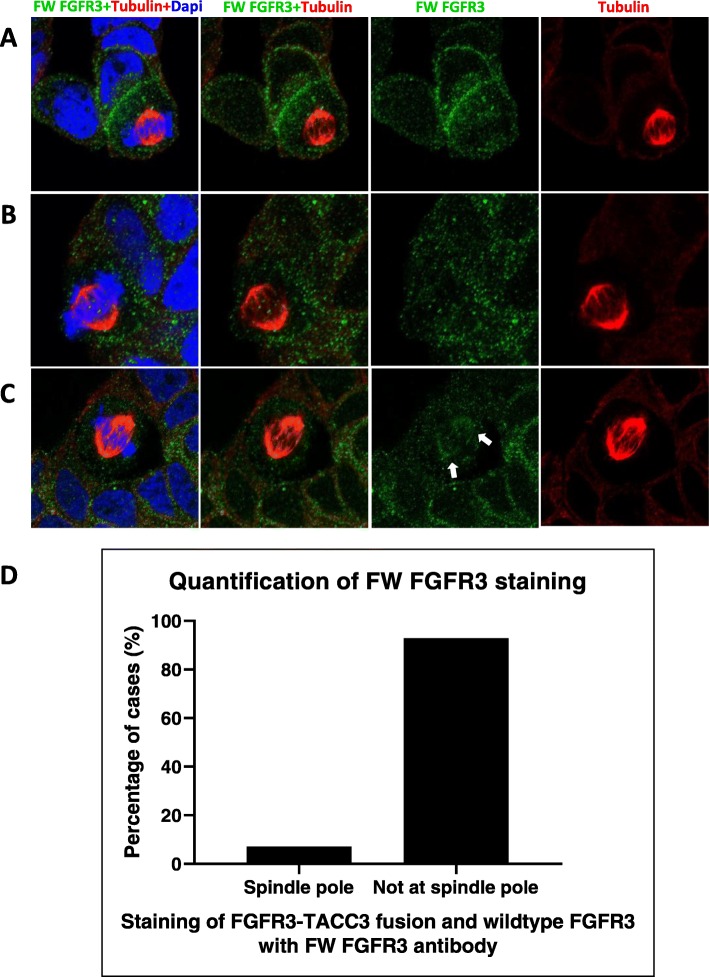
Immunofluorescent staining of mitotic SUM185PE cells with the FW FGFR3 antibody. **a**-**c**, Imaging was undertaken as in Fig. 3c-d, except that the FW FGFR3 antibody was used. **d**, Quantification of FW FGFR3 immunostaining localization. Images are representative of 3 biological replicates, each involving analysis of at least 10 cells

Use of the wildtype FGFR3 and FW FGFR3 antibodies for indirect immunofluorescent imaging revealed immunoreactivity in the cytoplasm and plasma membrane (Fig. 3-3a-b). In addition, SUM185PE cells undergoing mitosis were co-stained with tubulin antibodies and the wildtype FGFR3 or FW FGFR3 antibodies (Fig. 3-3, 4-4c-d, a-c). SUM185PE cells stained with the wildtype FGFR3 antibody only showed localization at the cell membrane and in the cytoplasm (Fig. 3-3c-d). However, upon use of the FW FGFR3 antibody, while the vast majority of dividing SUM185PE cells exhibited immunostaining at the cell membrane and in the cytoplasm (Fig. 4-4, 4a-b, d), 2 out of 28 cells examined (7%) exhibited additional localization at the mitotic spindle (Fig. 4-4c-d). Given the data obtained using the wildtype FGFR3 antibody (Fig. 3-3c-d), this indicates that the additional staining must arise from the FGFR3-TACC3 fusion. Overall, these data indicate that the previously reported localization of FGFR3-TACC3 to the mitotic spindle [35] occurs, but is not a common event in this TNBC model.

### Wildtype FGFR3 and the FGFR3-TACC3 fusion exhibit contrasting functional roles in SUM185PE cells

**Fig. 5:**
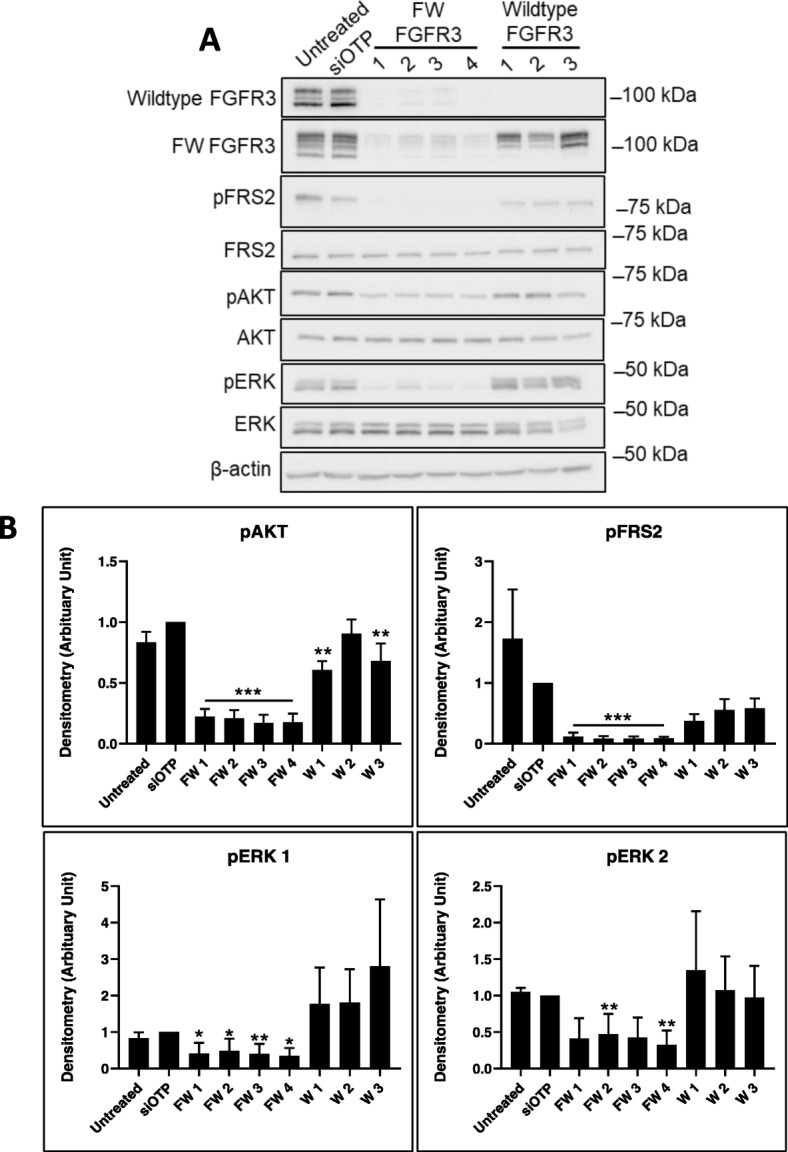
Effect of FGFR3 knockdown on downstream signaling in SUM185PE cells. **a**, SUM185PE cells were reverse transfected with 20 nM of individual siRNAs targeting FGFR3-TACC3 fusion and wildtype FGFR3 (FW FGFR3 1–4), or wildtype FGFR3 only (W1–3), and the indicated downstream signaling proteins analysed by Western blot. **b**, Quantification by densitometry of (A). Data were first normalized relative to the β-actin loading control, then phosphorylated proteins were normalized relative to total protein, then data were expressed relative to the siOTP control which was arbitrarily set at 1.0. Error bars: mean ± standard error, of three biological replicates. * indicates *p*-value of < 0.05, ** < 0.01, *** < 0.001

**Fig. 6:**
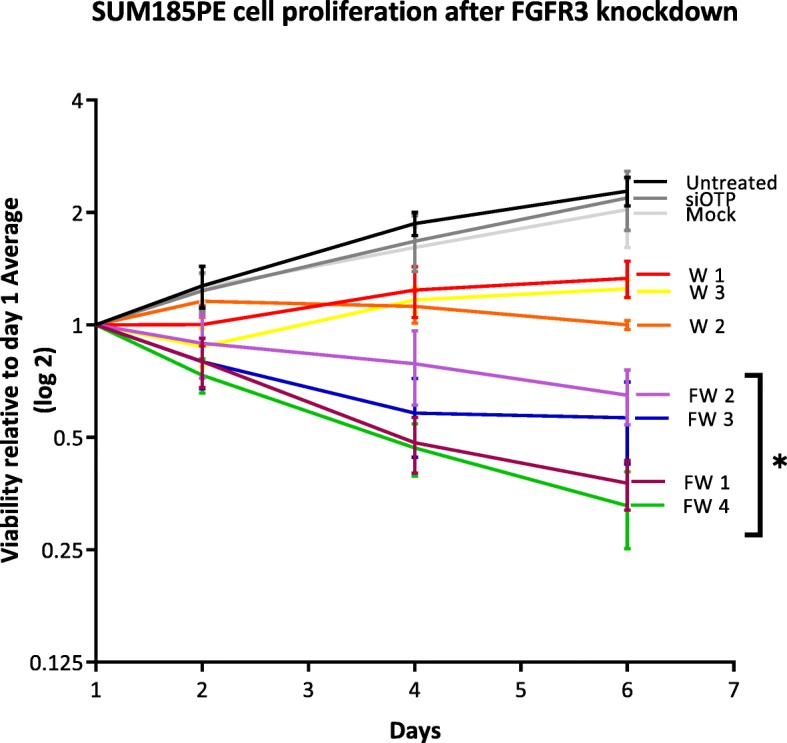
Effect of FGFR3 knockdown on SUM185PE cell proliferation. SUM185PE cells were reverse transfected with 20 nM of individual siRNAs targeting FGFR3-TACC3 fusion and wildtype FGFR3 (FW 1–4), or wildtype FGFR3 only (W1–3) and cell proliferation indirectly assayed via MTS absorbance. Error bars: mean ± standard error, of three biological replicates. W1, W2 and W3 were associated with *p*-values of 0.17, 0.07, and 0.13, respectively. * indicates *p*-value of < 0.05

To characterize the contribution of wildtype FGFR3 and the FGFR3-TACC3 fusion, knockdowns were undertaken with siRNAs that target both the FGFR3-TACC3 fusion and wildtype FGFR3 (FW FGFR3) or only wildtype FGFR3. Knockdown of both FGFR3-TACC3 fusion and wildtype FGFR3 expression decreased phosphorylation of the downstream signaling proteins FRS2, AKT and ERK, and induced cell death in SUM185PE cells (Figs. 5-6). In contrast, knockdown of wildtype FGFR3 reduced activation of AKT, but not FRS2 and ERK, and resulted in a trend for decreased cell proliferation (Figs. 5-6).

**Fig. 7:**
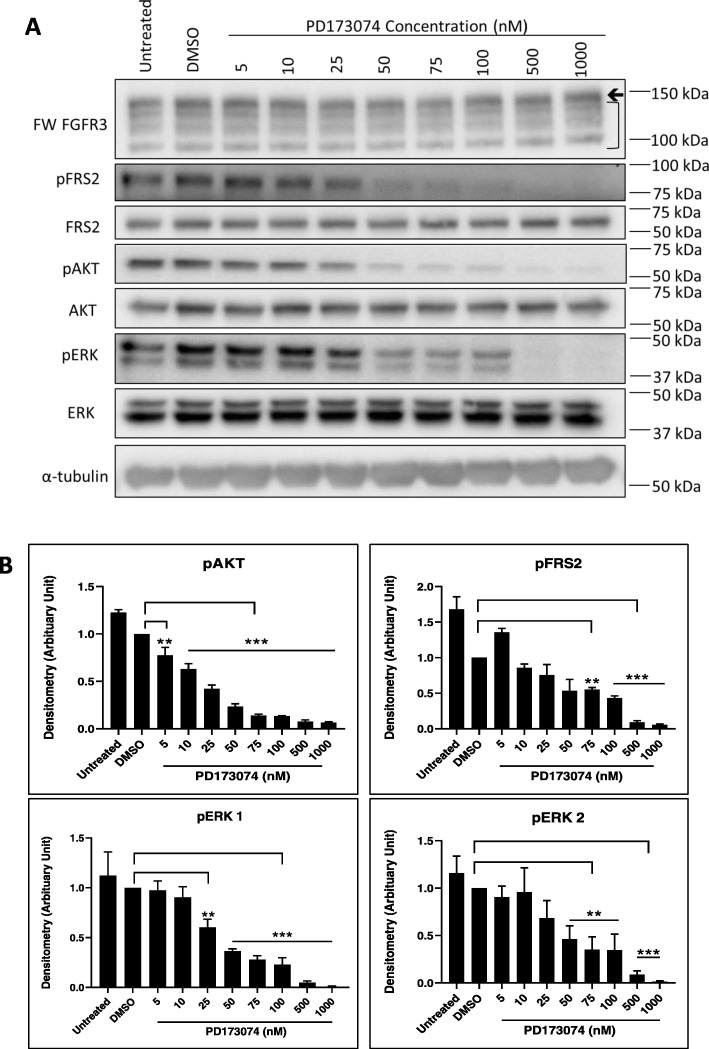
Dose dependent effect of the FGFR inhibitor PD173074 on FGFR3 downstream signaling pathways in the SUM185PE cell line. **a**, Expression/activation of downstream signaling proteins 1 h post-treatment with the indicated doses of PD173074. Arrow indicates FGFR3-TACC3 fusion protein, bracket indicates wildtype FGFR3. **b**, Quantification by densitometry of (A). Data were first normalized relative to the tubulin control, then phosphorylated proteins were normalized to total protein, finally data were expressed relative to the DMSO control which was arbitrarily set at 1.0. Error bars: mean ± standard error, of three biological replicates. ** indicates *p*-value of < 0.01,*** < 0.001

In order to further evaluate these forms of FGFR3 as potential therapeutic targets, we also determined the effects of the small molecule inhibitor PD173074 on signaling and proliferation. This is an ATP-competitive and type-I inhibitor, which targets FGFR1–3 and to a lesser extent, VEGFR2. It has a similar binding mode to other FGFR inhibitors that are in clinical trials (e.g. Erdafitinib, BGJ398, Pemigatinib and Dovitinib). Its selectivity for FGFR1–3 is similar to that of BGJ398 and Pemigatinib, but is much greater than that of Dovitinib, which is a multikinase inhibitor that also targets VEGFRs, PDGFR-β, c-kit and FLT3 and is likely to elicit differing biological effects [46, 47]. Treatment of SUM185PE cells with 5–75 nM PD173074 for 1 h led to a significant reduction in the phosphorylation of AKT, ERK1/2 and FRS2, with AKT phosphorylation being the most sensitive to drug treatment (Fig. 7).

**Fig. 8:**
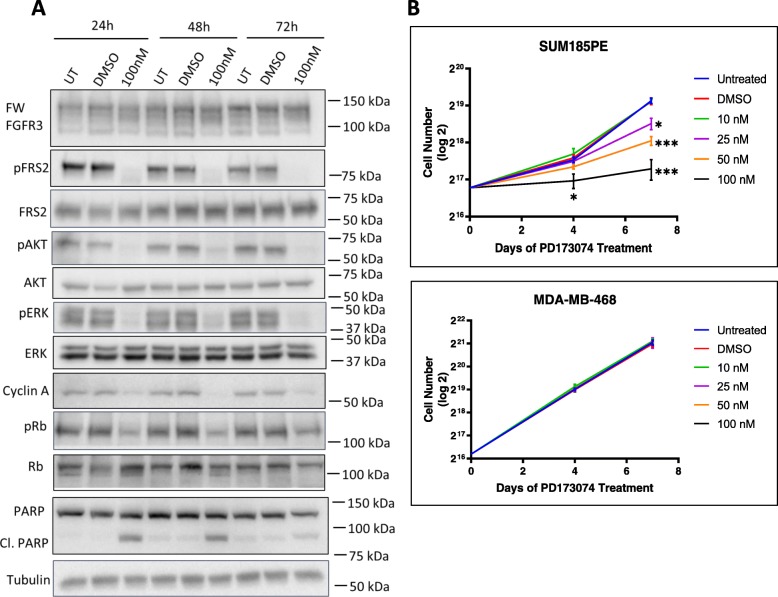
Effect of PD173074 on proliferation and apoptosis in SUM185PE cells. **a**, Effect on cell cycle and apoptosis markers. SUM185PE cells were treated with PD173074 for 24 h, 48 h and 72 h and the effect on the indicated proteins analysed by Western blotting. UT indicates ‘untreated group’ as a control for DMSO addition, in order to monitor any effect of DMSO on cell cycle regulators. **b**, Effect of PD173074 on proliferation of SUM185PE and MDA-MB-468 cells. Cell proliferation was determined by direct cell counting. Error bars: mean ± standard error, of three biological replicates * indicates *p*-value of < 0.05, *** < 0.001

In addition, administration of PD173074 for 24–72 h resulted in decreased expression of Cyclin A and pRb, and detection of cleaved PARP (Fig. 8a). Treatment with PD173074 also decreased SUM185PE cell proliferation in a dose-dependent manner, while no effect was observed in the negative control cell line MDA-MB-468 (Fig. 8b). Overall, these data indicate that the FGFR3-TACC3 fusion, and not wildtype FGFR3, is the main oncogenic driver in SUM185PE cells, and that targeting this oncoprotein leads to cell cycle arrest in the G1 phase of the cell cycle and also apoptosis.

### Functional role of FGFR3 in TNBC cell lines with low to moderate levels of FGFR3 phosphorylation

**Fig. 9:**
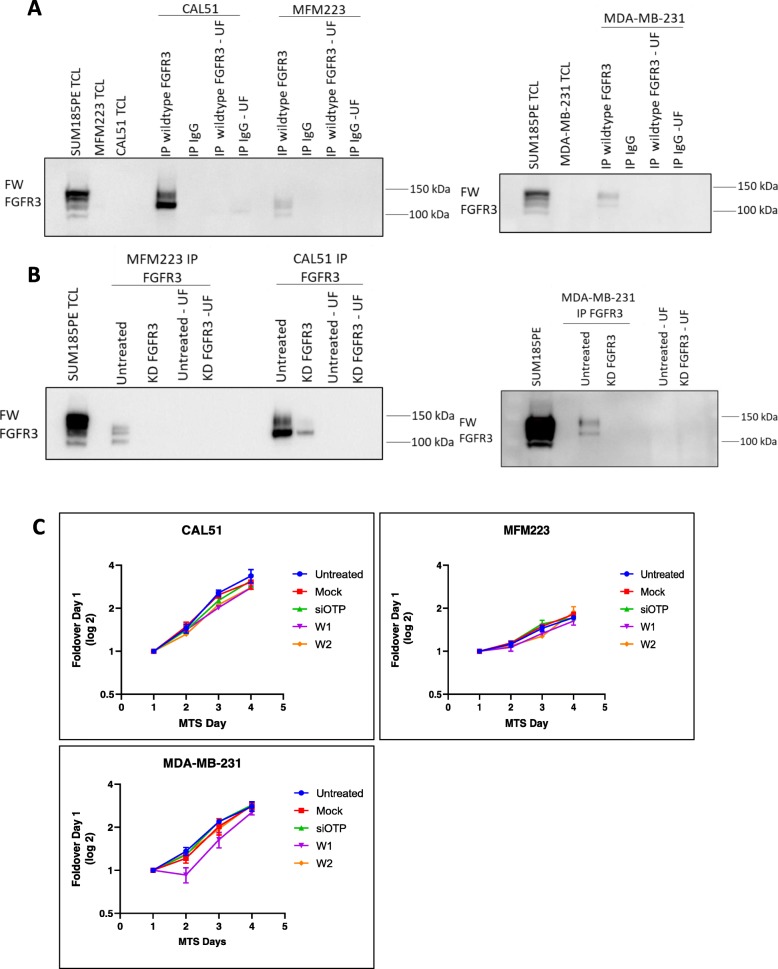
Expression and function of FGFR3 in TNBC cell lines exhibiting low-moderate FGFR3 phosphorylation. **a**, FGFR3 expression analysed by immunoprecipitation and Western blot. Lysates from CAL51, MFM223 and MDA-MB-231 cells were subjected to immunoprecipitation of wildtype FGFR3, which was then detected by Western blotting using the FW FGFR3 antibody. IgG was used as a negative control for immunoprecipitation. TCL = total cell lysate. UF = unbound fraction. **b**, Confirmation of FGFR3 expression by knockdown. Cell lines from (A) were subjected to FGFR3 knockdown prior to immunoprecipitation and Western blot analysis. KD = knockdown. **c**, Effect of FGFR3 knockdown on cell proliferation. Cells were transfected with 20 nM of individual siRNAs targeting wildtype FGFR3 (W1–2) and cell proliferation indirectly assayed via a MTS absorbance assay. Error bars: mean ± standard error, of three biological replicates

Three cell lines exhibited low to moderate FGFR3 phosphorylation in the phosphoproteomic dataset on sites specific to FGFR3: MDA-MB-231, MFM223 and CAL51 (Fig. 1a). Since FGFR3 was undetectable in these cells by direct Western blot (Fig. 1b, Fig. 9a), lysates were subjected to immunoprecipitation to enrich for FGFR3 and the receptor detected by Western blot using the FW FGFR3 antibody (Fig. 9a). This confirmed that each of these cell lines indeed expresses FGFR3, with the identity of the receptor validated by siRNA knockdown (Fig. 9b). However, FGFR3 knockdown did not significantly affect cell proliferation in any of the cell lines (Fig. 9c), indicating that the oncogenic role of FGFR3 in TNBC is likely limited to contexts where it is hyperactivated due to mutation or gene translocation events.

### Evaluation of FGFR3 alterations in breast cancer patients using public datasets

**Fig. 10:**
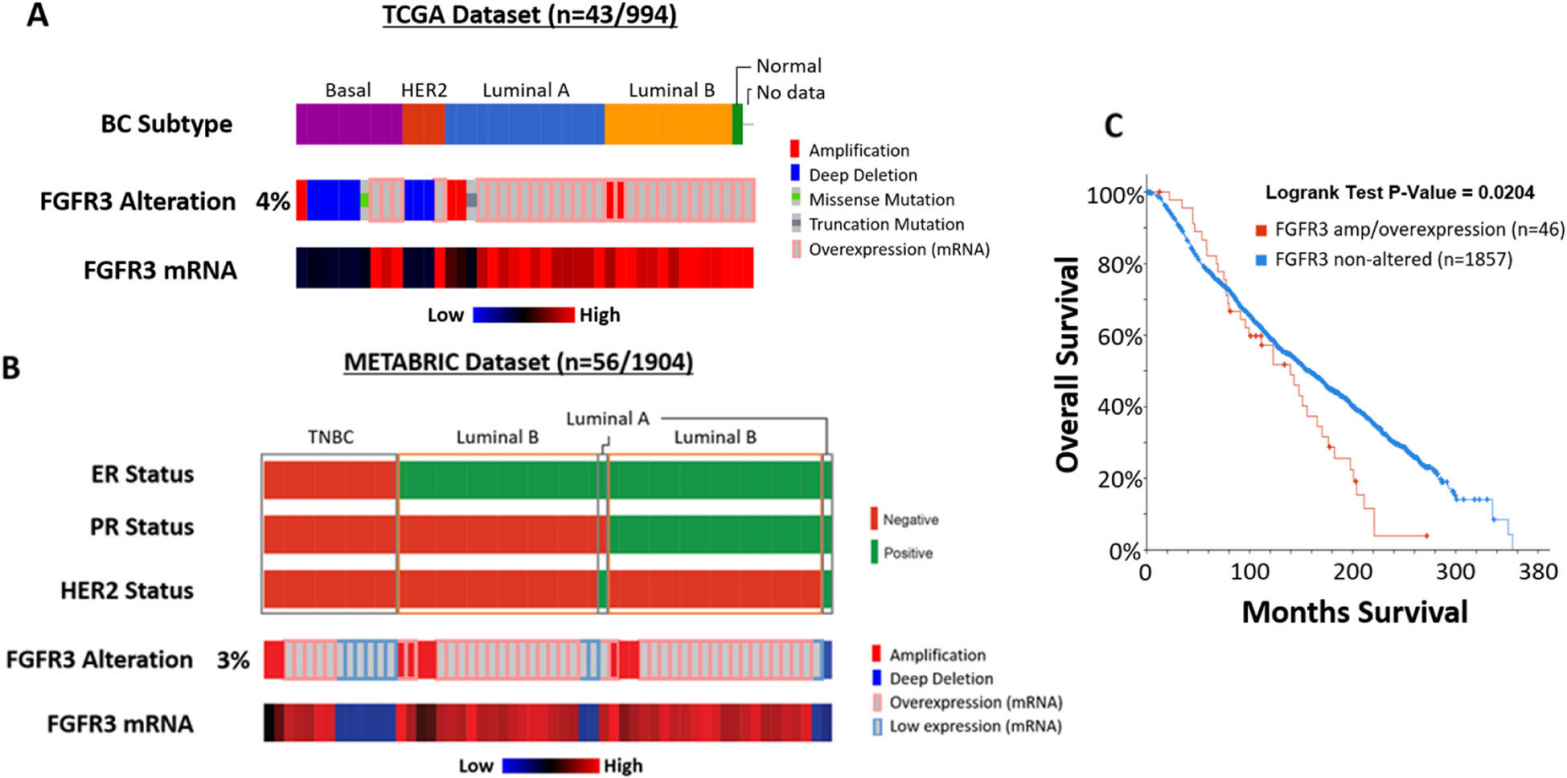
FGFR3 alterations in human breast cancer. Frequency of FGFR3 alterations in breast cancer patients analysed using two breast cancer patient datasets, **a**, Pan-cancer Atlas dataset from TCGA and **b**, METABRIC dataset, using cBioportal (note that no mutation data are available for the METABRIC dataset). Only patients with FGFR3 alterations are displayed for brevity. For both cohorts, the breast cancer subtypes based on ER/PR and HER2 receptor status are displayed. **c**, A Kaplan–Meier plot showing patients with amplification and/or overexpression of FGFR3 (*n* = 46) are significantly associated with worse overall survival compared to those without these alterations (*n* = 1857) in the METABRIC dataset. A Logrank test was used, *P*-value = 0.0204 (*P*-value < 0.05 considered significant). Survival data for the two patient groups were extracted and downloaded from cBioportal, and survival analysis performed using in-house Matlab script

The TCGA and the Metabric datasets were analyzed using cBioportal to determine the frequency of FGFR3 alterations in terms of overexpression, mutation, amplification and deletion in different breast cancer subtypes. In the TCGA and Metabric datasets, 43 out of 994 (4%) and 56 out of 1904 (3%) of breast cancer patients have FGFR3 alterations, respectively (Fig. 10-10a-b). FGFR3 amplification, which affected 5 breast cancer patients (0.5%) and 9 cases (0.5%) in the TCGA and Metabric datasets respectively, was observed in the TNBC/basal, luminal A and luminal B subtypes, with FGFR3 deep deletion mostly detected in the TNBC/basal or HER2+ subtypes (Fig. 10-10a-b). FGFR3 overexpression was more common in luminal subtypes than TNBC/basal. In the Metabric dataset, breast cancer patients with amplified and/or overexpressed FGFR3 (46 out of 1903, 2%) have a significant (p-value of 0.0204) worse overall survival compared to breast cancer patients without FGFR3 alterations (Fig. 10c). These data confirm that potentially oncogenic FGFR3 alterations do occur in TNBC, as well as other breast cancer subtypes, albeit at low frequency.

## Discussion

FGFR signaling has many biological roles in normal physiology, including regulation of cell proliferation, migration and survival, however in breast cancer progression, FGFR signaling is often deregulated [24, 33]. FGFR1 amplification is the most common aberration, followed by FGFR2 amplification, and the roles of these receptors have been characterized in detail [23, 28, 30]. To date, our work is the most detailed study on FGFR3, describing its activation, expression and function in TNBC.

Our characterization of FGFR3 function in TNBC cell lines exhibiting differing levels of receptor activation demonstrated that only the aberrantly activated FGFR3-TACC3 fusion in SUM185PE cells functioned as an oncogenic driver, at least in vitro. This fusion is constitutively activated due to dimerization driven by the TACC3 region [35, 38].

Knockdown of wildtype FGFR3 in SUM185PE cells resulted in modest effects on AKT activation and cell proliferation, while having no effect on MFM223, CAL51 and MDA-MB-231 cell proliferation. Since expression of wildtype FGFR3 is higher in SUM185PE cells than the other cell lines, this suggests that a threshold level of expression/activation is required for detectable effects on signaling and proliferation. However, other factors that likely limit the biological role of FGFR3 in TNBC cell lines are the genetic background of the cells, and production of autocrine ligands. MFM223 cells exhibit FGFR2 amplification, which may make FGFR3 redundant. CAL51 cells express detectable FGFR1 and FGFR2 as well as autocrine FGF2 and are sensitive to PD173074 [33]. Therefore, these data and our phosphoproteomic and functional analyses, indicate that FGFR1 and FGFR2 must play a more important functional role in these cells, rather than FGFR3. However, MDA-MB-231 cells are resistant to PD173074 and express very low levels of FGF2 [33] that will limit activation of expressed FGFRs. In light of the latter finding, it remains possible that the oncogenic potential of FGFR3 may be different in vivo, where cancer cells are exposed to paracrine FGFs from the stroma.

This report is the first study of FGFR3-TACC3 signaling and localization in the context of breast cancer. Consistent with previous studies on head and neck malignancies [37] and glioblastoma [35, 48], attenuation of FGFR3-TACC3 activation decreased phosphorylation of FRS2, AKT and ERK. However, while in glioblastoma, the FGFR3-TACC3 fusion reportedly localizes to the mitotic spindle poles [35], we observed that the vast majority of FGFR3-TACC3 fusion and all of wildtype FGFR3 localized to the cytoplasm and plasma membrane, consistent with data from HeLa cells [38], the requirement for entry into the secretory pathway or localization to the plasma membrane for FGFR3-TACC3 oncogenic function [45] and coupling of FGFR3-TACC3 to canonical downstream signaling pathways usually activated at the plasma membrane. That said, the occasional detection of FGFR3-TACC3 at the spindle poles indicates that this still represents a potential mechanism whereby this oncoprotein may contribute to tumor progression, for example by promoting aneuploidy in a small subpopulation of cells [35].

In the TCGA and Metabric datasets, FGFR3 alterations are observed in a total of 99 out of 2898 breast cancer patients (3.4%), with 16 out of 2898 (0.6%) cases reflecting FGFR3 amplification or mutation (Fig. 10-10a-b). Other studies support the presence, albeit at low frequency, of FGFR3 alterations in breast cancer. In a study of 182 ER+ breast cancer patients, FGFR3 was mutated in 3 out of 126 (2.4%) primary samples and 1 out of 57 (1.8%) metastatic samples [49]. In addition, a FGFR3-TACC3 fusion was detected in 1 out of 253 TNBC tumors (0.4%) [34]. Despite low frequencies, therapeutic targeting of FGFR3 represents a potential option for cancers exhibiting oncogenic forms of FGFR3, supported by our data regarding the efficacy of PD173074 in SUM185PE cells.

In addition to FGFR3 amplification, deep deletions of FGFR3 occur (Fig. 10-10a-b). This has also been observed in inflammatory breast cancer, where 10 out of 156 (6.4%) cases had FGFR3 deletion [50]. The loss of FGFR3 is significantly associated with higher grade urothelial bladder tumors [51] and also leads to chondroma-like lesion formation by downregulating ERK signaling whilst upregulating Hedgehog signaling, suggesting tumor suppressive roles of FGFR3 [52]. Furthermore in pancreatic cancer, where FGFR3 expression is downregulated, FGFR3 functions as a tumor suppressor in cancer cells of epithelial phenotype and an oncogene in cells of mesenchymal phenotype, highlighting context-dependent functional roles [53]. Despite the presence of FGFR3 deletions in a subset of breast cancer patients, amplification and/or overexpression of FGFR3 is associated with poor prognosis in the Metabric dataset, and an immunohistochemical study in breast cancer also identified FGFR3 as a negative prognostic factor [54]. Consequently, while the presence of FGFR3 deletions raises the possibility of context-dependent tumor suppressor roles in a subset of breast cancers, strong evidence also exists for a positive role for this receptor in breast cancer progression.

## Conclusions

Increased expression and activation of FGFR3 occurs in TNBC but an oncogenic role could only be demonstrated for a rare example of a FGFR3-TACC3 fusion. These results indicate that targeting FGFR3 may represent a therapeutic option for TNBC, but only for a select group of patients with oncogenic FGFR3 alterations.

## Supplementary information

**Additional file 1: Figure S1.** Interactions of proteins exhibiting tyrosine phosphorylation specific to SUM185PE cell line using STRING software

**Additional file 2: Table S1.** Quantifiable FGFR tyrosine phosphorylated peptide expression (log2) identified across 24 TNBC cell lines **Table S2.** Quantifiable tyrosine phosphorylated peptide expression (log2) specific to SUM185PE cell line. Tyrosine phosphorylation of TACC3 (highlighted in pink) only occurs in SUM185PE

## Data Availability

All data generated or analysed during this study are included in this published article and its supplementary information files. The original data supporting these findings are available at any time upon request to the corresponding author.

## Abbreviations

DDA: Data-dependent acquisition
ER: Estrogen receptor
FDR: False discovery rate
FGFR3: Fibroblast growth factor receptor 3
FRS2: Fibroblast growth factor receptor substrate 2
HER-2: Human epidermal growth factor receptor-2
HRM-DIA: Hyper-reaction monitoring data-independent acquisition
iRT: Indexed Retention Time
LC-MS/MS: Liquid chromatography-tandem mass spectrometry
MS: Mass-spectrometry
PR: Progesterone receptor
RTK: Receptor tyrosine kinase
TACC3: Transforming acidic coiled-coil protein 3
TCGA: The cancer genome atlas
TNBC: Triple negative breast cancer
