# Supplementary material for: FGFR3 signaling and function in triple negative breast cancer
Source: Cell Commun Signal. 2020 Jan 27;18:13. doi: 10.1186/s12964-019-0486-4 (PMC6986078; doi:10.1186/s12964-019-0486-4)
Supplement: Supplementary file 2 — Additional file 1: Figure S1. Interactions of proteins exhibiting tyrosine phosphorylation specific to SUM185PE cell line using STRING software [file 12964_2019_486_MOESM2_ESM.pdf]

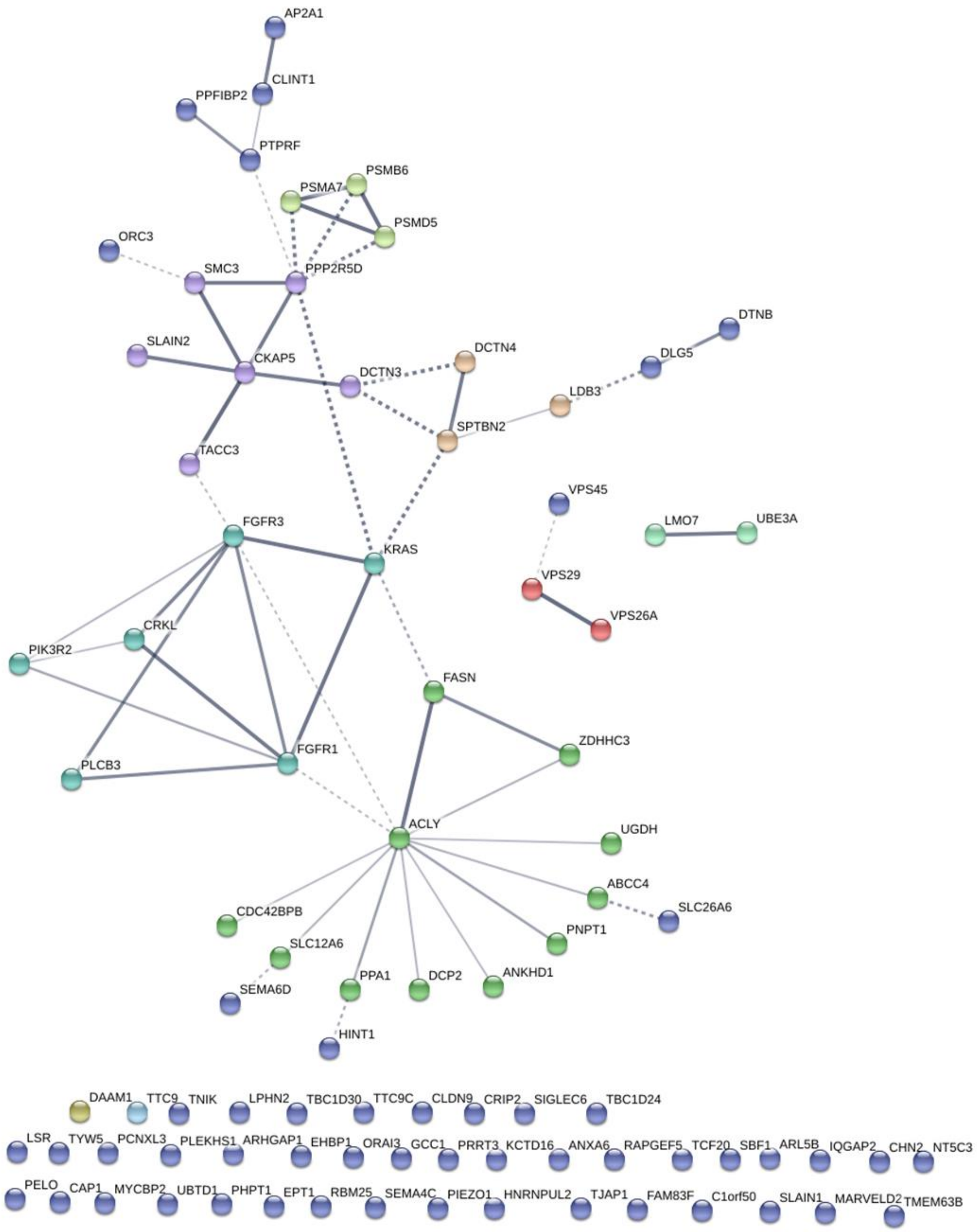

Supplementary Figure 1: Interactions of proteins exhibiting specific tyrosine phosphorylation specific to SUM185PE cell line using STRING software.
